# Mesomorphic, Computational Investigations and Dyeing Applications of Laterally Substituted Dyes

**DOI:** 10.3390/molecules27248980

**Published:** 2022-12-16

**Authors:** Hoda A. Ahmed, Mohamed A. El-Atawy, Fowzia S. Alamro, Nada S. Al-Kadhi, Omaima A. Alhaddad, Alaa Z. Omar

**Affiliations:** 1 Department of Chemistry, Faculty of Science, Cairo University, Cairo 12613, Egypt; 2 Chemistry Department, Faculty of Science, Taibah University, Yanbu 46423, Saudi Arabia; 3 Chemistry Department, Faculty of Science, Alexandria University, P.O. Box 426 Ibrahemia, Alexandria 21321, Egypt; 4 Department of Chemistry, College of Science, Princess Nourah bint Abdulrahman University, P.O. Box 84428, Riyadh 11671, Saudi Arabia; 5 Chemistry Department, College of Sciences, Taibah University, Al-Madina 30002, Saudi Arabia

**Keywords:** liquid crystals, azo dye, color strength, DFT, fastness properties

## Abstract

Two groups of laterally substituted non-mesomorphic and liquid crystalline materials bearing monoazo group were prepared and investigated via experimental and theoretical techniques. The molecular structures of the designed dyes were evaluated by FT-IR and NMR spectroscopic analyses. Mesomorphic examinations for all synthesized dyes were investigated by polarized optical microscopy (POM) and differential scanning calorimetry (DSC). Results revealed that, the thermal and optical properties of investigated compounds are mainly dependent on their molecular geometry. The optimized geometries of the azo derivatives and their electronic absorption of the dyes were carried out using the B3LYP/6-311G level of the DFT method. The azo dyes were measured for their dyeing performance on polyester fabrics. The dyed fabrics have excellent fastness properties with a color strength of 1.49–3.43 and an exhaustion rate of 82–64%. The chemical descriptor parameters of disperse azo dyes in gas phase were calculated and correlated with dyeing parameters.

## 1. Introduction

The synthetic colors have broad applications in different fields of modern industry, such as leather, textiles, papers, hair dye, and the industry of food [1,2]. The color intensity and the azo dyes interactions with fibers depend on the chemical formula and the structure of both the compound that is used as a dye and the dyed fabrics [3,4,5,6,7,8,9,10]. Non-ionic dyes with an azo chromophore are commonly used on hydrophobic fabrics such as polyester, cellulose acetate, and nylon [11].

Liquid crystalline materials offer a wide range of technical applications, including optical displays, emitting diodes, and organic photoconductors [12,13,14,15,16,17,18,19,20,21]. Geometrical–characteristic relationships are a useful tool for synthesizing appropriate structural shapes and achieving desirable qualities for specific industrial applications [12,13,14,15,16,17,18,19,20,21]. Molecular shape allows for significant changes in mesomorphic characteristics and plays a key influence in the formation, type, and thermal stability of the mesophase formed [22,23]. Furthermore, in the development of thermotropic liquid crystals (LCs) for proper characteristic applications, the choice of laterally attached groups, terminal wings, and mesogenic linkages are important criteria.

Because of their unique optical properties, liquid crystals (LCs) have attracted a lot of attention as a branch of intelligent materials and have played important roles in theoretical and experimental research as well as engineering applications [24,25]. Calamitic liquid crystals have already been known for their ability to efficiently reflect circularly polarized incident light [26,27]. Red, orange, yellow, green, blue, indigo, and purple are the colors that change in daylight as the temperature rises; the opposite is true as the temperature is lowered. Modifying the molecular geometries of LC compounds can change their thermal and optical properties. To design new liquid crystalline materials, it is necessary to understand the relationship between mesogens’ molecular structures and their mesomorphic properties. Several calamitic azo/ester LCs derivatives have been studied, and they are frequently examined due to their interesting optical features [26,27,28,29].

**Scheme 1:**
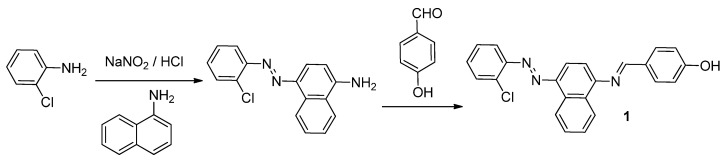
Synthesis of azo Schiff base **1**.

**Scheme 2:**
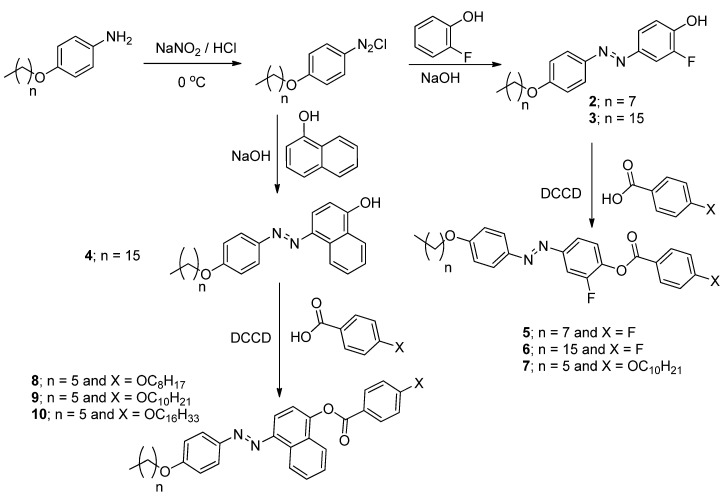
The synthetic pathway of liquid crystal disperse azo dyes **2**–**10**.

The main target of this work is to synthesize mono azo and Schiff base phenols as liquid crystals with different polar lateral and terminal substituents **1**–**10**, Scheme 1 and Scheme 2. All designed derivatives will be investigated thermally and mesomophically using DSC and POM. Density functional theory (DFT) was used to analyze the experimental mesomorphic properties in terms of predicted parameters. Furthermore, application of synthesized dyes on polyester fibers and evaluation of their fastness properties as well as exhaustion rate and color strength were investigated.

## 2. Results and Discussion

### 2.1. Thermal and Mesomorphic Properties

**Figure 1:**
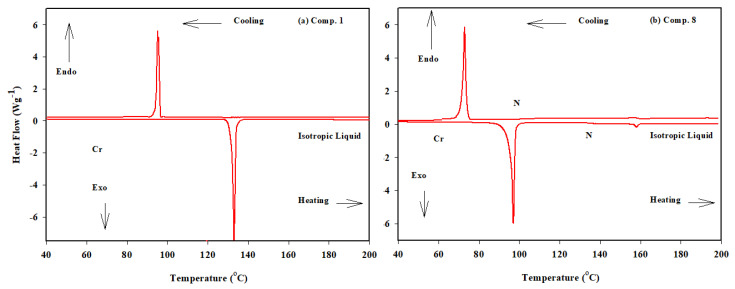
DSC thermograms at a heating rate of 10 °C/min of compounds (**a**) **1** and (**b**) **8** from the second heating and cooling scan.

**Figure 2:**
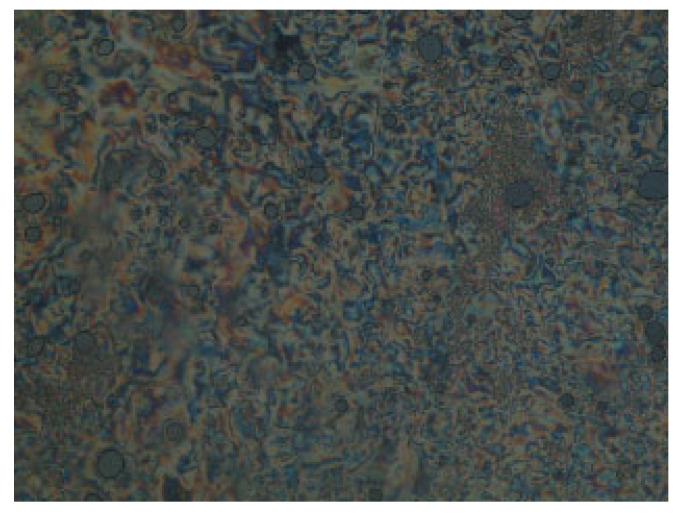
Nematic pattern under POM of compound **8** at 130.0 °C upon heating.

**Figure 3:**
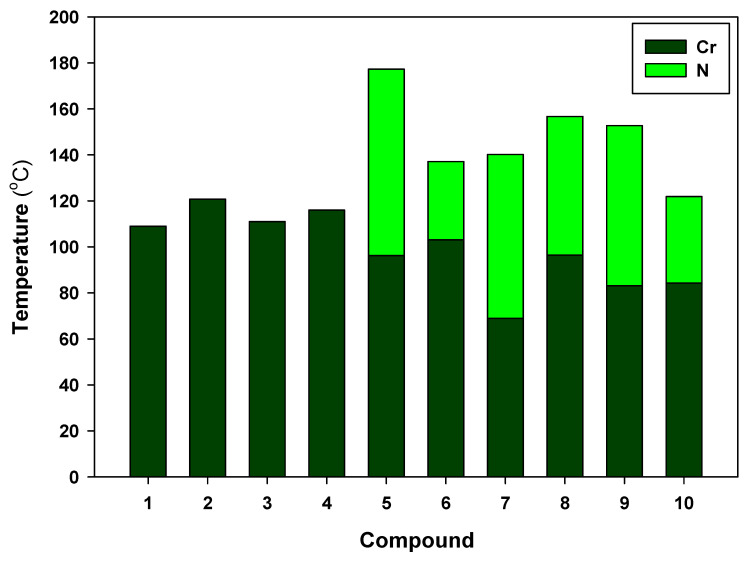
DSC transitions for investigated compounds **1**–**10**.

Table 1 summarizes the transition temperatures of synthesized azo derivatives **1**–**10** as well as their corresponding enthalpies, as determined by DSC measurements. Moreover, polarized optical microscopy was used to identify their mesophases. As examples, DSC thermograms for derivatives **1** and **8** are shown in Figure 1 on heating and cooling cycles. DSC measurements were taken in second heating scanning to ensure the thermal stability of the examined azo compounds. In addition, the POM textures are consistent with the findings of DSC examinations, Figure 2. A graphical transition temperature of DSC data was provided in Figure 3 to compare the mesomorphic properties of the synthesized derivatives **1**–**10**.

As can be seen from Table 1 and Figure 3, all laterally substituted phenolic derivatives, **1**–**4**, showed only one transition peak during heating and cooling cycles, as indicated by DSC thermograms assigned to the Cr-to-isotropic liquid phase (during heating) in addition to the isotropic-to-Cr phase (during cooling). Moreover, all laterally substituted three-ring compounds, **5**–**10**, have two transition peaks upon heating/cooling rounds of DSC thermograms, which were ascribed to the Cr–nematic and nematic–isotropic liquid mesophase on heating and reversed upon cooling scan. These findings are consistent with POM studies. According to Table 1 and Figure 3, also, the laterally azo derivatives **5**–**10** have monomorphic nematic phase enantiotropically. Furthermore, the melting transition temperature is mainly depending on the molecular structure of designed compound [30].

Due to its tiny size, the fluorine atom can be easily incorporated into mesomorphic structures without causing any steric disruption. As a result, LCs mesophases can still be observed as in dyes **5**–**7** [31]. The optical morphology, transition events, and other physical properties are all enhanced by the high polarity. Furthermore, depending on its location and orientation in the molecule, the polar terminal fluorine alters the polarizability and dipole moment of the entire molecular structure. This is evident in the examined molecule’s mesophase and optical properties. In addition, the incorporation of the naphthyl group to the central molecule disrupts the smectic molecular packing and giving only the nematic mesophase (**8**–**10**) [24,32]. As a result, the insertion of a lateral group in the molecule causes a minor change in the molecular structure, but the polarity and orientation of the dipole moments are changed. According to earlier reports [24,32], the type of the phase and its stability are mostly determined by the dipole moment of the mesogenic component of the molecule, which varies depending on the polar groups present and its steric impact, which subsequently varies based on the volume and location of the substituent. Moreover, adding terminals to an LC material has two opposing effects: first, a decrease in phase stability due to the steric influence of the terminal substituent [33,34], and second, an increase or decrease in molecular anisotropy depending on the polarizing effect of the substituent. The position and polarity of the coupled lateral and terminal groups determine the dipole moment of the entire compound. Furthermore, the terminal and lateral moieties are important in determining the melting temperatures of the synthesized derivatives.

### 2.2. Electronic Absorption Spectra and Substituent Effect

**Figure 4:**
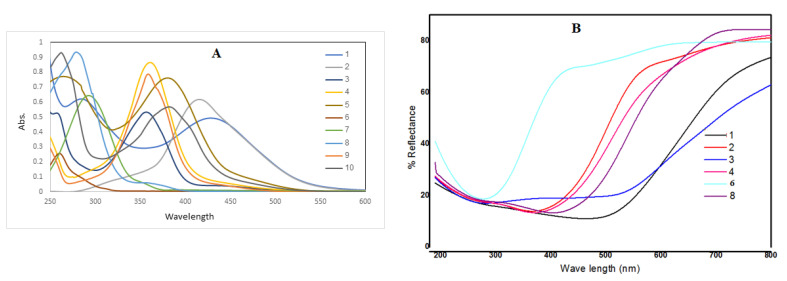
(**A**) Absorption spectra of azo dyes **1**–**10** in chloroform, (**B**) Reflectance curves of the dyed polyester fabrics.

The absorption maxima **λ_max_** and the observed colors are listed in Table 2. The experimental results showed that **λ_max_** are ranged in the region of 260–430 nm, Figure 4, whereas the computed absorption maxima are in the range 306.63–374.47 nm.

Table 2 showed that dye **1** has the greatest λ_max_ among all the prepared azo dyes **1**–**10**; this bathochromic shift is ascribed to the greater conjugation of azomethine with azo groups through naphthyl moiety in dye **1**. Because of the conversion of the phenolic moiety into a benzoate ester, dyes **6** and **7** exhibited a hypsochromic shift compared to their precursors, dyes **2** and **3**. This pattern of behavior indicates that the action of the substituent has only a mild influence on the absorption values.

### 2.3. Dyeing Process and Fastness Properties

Selected disperse azo dyes **1**–**4**, **6**, and **8** were applied to knitted polyester fabrics namely, polyethylene terephthalate (PET) through high temperature dyeing method at 130 °C with ratio 1:20 of material to liquor. An amount of 1% dye was used for dying (calculated on weight of the polyester)**.**

The color fastness results of azo dyes **1**–**4**, **6**, and **8** including washing, perspiration (acidic and alkaline), scorch (cotton and polyester), and light fastness have been found using standard procedure [6,7] and are presented in Table 3.

All of the measured properties were assessed with gray scale from 1—poor to 5—excellent except light fastness from 1 to 8 [8]. The results in Table 3 indicated that the disperse azo dyes **1**–**4**, **6**, and **8** on polyester fabrics have good to excellent fastness properties. Dyes **1** and **4** displayed the highest fastness levels for polyester fabrics. Azo dyes **1**–**4**, **6**, and **8** have good fastness levels to alkaline perspiration and moderate to acidic perspiration. In a similar way, the dyes displayed good fastness to polyester scorch with respect to cotton scorch except dye **6** in which cotton scorch is better. Furthermore, the light fastness is better in dyes **1**, **2**, **6**, and **8** and weak in dyes **3** and **4**.

### 2.4. Dye Exhaustion, Reflectance, and Color Strength

The consumption of the azo dyes by polyester fiber was measured by sampling the dye bath before and after the dyeing process. The exhaustion percentage (%E) of each of the dyes is determined by comparing the results of spectrophotometric measurements taken of the concentration of dye bath solution before (C_1_) and after dyeing (C_2_). The data in Table 4 indicated that the azo dyes **1**–**4**, **6**, and **8** exhibited relatively moderate to good exhaustion (%); dye **1** displayed the highest exhaustion rate (82%), while dye **6** showed the lowest dye exhaustion (46%).

(1) $$\left(\%E=\frac{C_{1}-C_{2}}{C_{1}}100\right)$$

The color strength of the dyed polyester fabrics, Table 4, was determined in terms of K/S values at **λ_max_** using the Kubelka-Munk [10] Equation (2):

(2) $$K/S={\left(1-R\right)}^{2}/2R$$

where *R* is the decimal fraction of the reflection of the dyed fabric; *K* is the absorption coefficient; and *S* is the scattering coefficient.

The data in Table 4 reveal that the color strengths (K/S) of the dyed polyester fabrics lie in the range 1.49–3.43, indicating that the color strength depends mainly on the type of functional groups surrounding the azo group. This is apparently noticed from the hues of the fabrics treated with azo dyes **1**–**4**, **6**, and **8**, which vary from gray to brick red. Additionally, dyed polyester fabrics with dye **1** showed the highest K/S value while azo dye **6** exhibited the lowest color strength value.

### 2.5. Theoretical Study and the Molecular Descriptors

The density function theory (DFT) can be employed as a “green dyeing technique” because it works theoretically, and it can be considered as the easiest approach to study the molecular structure [10] and accordingly enable the researchers to study the dyeing mechanism. Chemical descriptor parameters [35,36,37,38] of the dispersed azo dyes **1**–**10** were computed using B3LYP/6-311G level to examine their dyeing performance, Table 5.

The energy of the highest occupied molecular orbitals **E_HOMO_** as well as the energy of the lowest unoccupied **E_LUMO_** of any chemical species are related to their ionization potential **IP** and electron affinity **EA** values [35,36,37,38,39]. Moreover, the global parameters including energy gap (**ΔE**) [40], electrophilicity index (**ω**), absolute electronegativity (**χ**), absolute hardness (**η**), absolute softness (**σ**), and chemical potential (**μ**) were calculated [41]. Generally, the binding capacity of a molecule improves with increasing the HOMO energy and decreasing the LUMO energy [42,43]. In other words, the binding capacity of the molecule increases as the **ΔE** value decreases. Subsequently, a small **∆E** value indicates high dyeing strength [11]. Higher values of chemical hardness (**η**) indicate less coloration of the fiber, whereas softness parameter (**σ**) indicates higher coloration intensity. It should be stated that strong color intensities should be accompanied with low electronegativity values for dyes [44,45]. Consequently, the dyeing strength increases with an increase in chemical potential. It should be taken into consideration that the previous descriptors deal only with the adsorption step of the dying process.

Table 5 points out that among the selected dyes used in the dyeing process, **1**–**4**, **6**, and **8**, dye **1** has the highest dyeing strength based on parameters ***η*** and ***S***, while dye **4** has the highest dyeing efficiency according to **E_HOMO_ µ** and **χ**. This matches with the experimental data of color strength for dyes **1** and **4**, which have the highest K/S values 3.43 and 2.75, respectively, and the presence of extra azomethine substituent helps the depth of dye **1** onto the fiber.

## 3. Materials and Methods

Materials and Methods are given in Supplementary Materials.

## 4. Conclusions

Experimental and theoretical techniques were used to study ten dyeing materials expressing liquid crystalline and non-mesomorphic behavior depending on their structures, **1**–**10**. FT-IR and NMR spectroscopy investigations were used to verify the molecular structures of the designed dyes. Polarized optical microscopy (POM) and differential scanning calorimetry (DSC) were used to examine the mesomorphic properties of all synthetic dyes. The electronic absorption spectra indicated that all dyes absorbed in the visible region except dye **6**, and this appeared from the gray color of the dyed polyester fabrics. The dyeing process performed on polyester fabrics and the results indicated that the disperse azo dyes have good to excellent fastness properties with relatively moderate to good exhaustion (%); dye **1** displayed the highest exhaustion rate (82%), while dye **6** is the lowest dye exhaustion (46%). Additionally, dyed polyester fabrics with dye **1** showed the highest K/S value (3.43), which is consistent with theoretical parameters.

## Figures and Tables

**Table 1:** Phase transition temperatures (°C), enthalpy of transition Δ*H*, kJ/mole and mesomorphic range (Δ*T*) for compounds **1**–**10**.

Comp.	*T*_Cr-I_	Δ*H*_Cr-I_	*T*_Cr-N_	Δ*H*_Cr-N_	*T*_N-I_	Δ*H*_N-I_	Δ*T*_N_
**1**	138.0	57.9	-	-	-	-	-
**2**	120.8	60.1	-	-	-	-	-
**3**	111.0	55.7	-	-	-	-	-
**4**	116.0	49.4	-	-	-	-	-
**5**	-	-	96.3	39.38	177.3	1.23	81.0
**6**	-	-	103.1	42.30	137.1	1.36	34.0
**7**	-	-	68.9	44.1	140.2	1.91	71.3
**8**	-	-	96.4	30.82	156.7	2.89	60.3
**9**	-	-	83.1	39.83	152.7	1.90	69.6
**10**	-	-	84.3	48.58	121.9	2.35	37.6

Cr-N = transition of solid to nematic phase. N-I = transition of Nematic to isotropic phase.

**Table 2:** Experimental and calculated absorption maxima of the azo dyes **1**–**10** in CHCl_3_.

Dye no.	Observed Color	Experimental (nm)	Calculated (nm)
**1**	yellow	430, 283	374
**2**	orange	408	307
**3**	orange	394	307
**4**	orange	389	341
**5**	yellow	390, 258	307
**6**	yellow	260	339
**7**	yellow	379	307
**8**	orange	383, 262	340
**9**	orange	403	340
**10**	orange	382, 262	339

**Table 3:** Fastness properties of selected disperse azo dyes **1**–**4**, **6**, and **8** on polyester fabrics.

Dye no.	Dyed PE Fabrics	Wash *	Perspiration **		Scorch ***		Light ****
			Acidic	Alkaline	Cotton	Poly Ester	
**1**	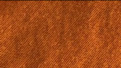	5	5	5	5	5	5–6
**2**	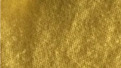	5	4–5	5	4-5	5	5–6
**3**	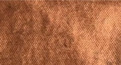	4–5	4–5	5	5	5	3–4
**4**	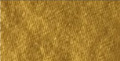	5	5	5	5	5	3–4
**6**	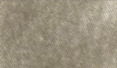	4–5	3–4	5	4–5	3–4	5–6
**8**	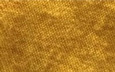	4–5	5	5	4–5	5	5–6

* AATCC16 (ISO 105 C06); ** AATCC15 (ISO 105 E04); *** AATCC13 (ISO 105 X11); **** AATCC 16 (ISO 105 B02).

**Table 4:** Dye exhaustion, reflectance, and color strength of azo disperse dyes on polyester fabrics at **λ_max_**.

Dye No	Dye Exhaustion	Reflectance (%)	K/S
**1**	82%	11.42	3.43
**2**	65%	13.80	2.69
**3**	54%	18.78	1.77
**4**	69%	13.54	2.75
**6**	46%	20.88	1.49
**8**	71%	13.59	2.74

**Table 5:** Chemical descriptor parameters of disperse azo dyes **1**–**10** in gas phase.

Parameter	1	2	3	4	5	6	7	8	9	10
**E_HOMO_** (**eV**)	−0.4649	−0.2876	−0.2875	−0.2643	−0.2928	−0.2726	−0.2912	−0.2705	−0.2704	−0.2707
**E_LUMO_** (**eV**)	0.0277	0.0497	0.0498	0.0481	0.0426	0.0400	−0.0447	0.0419	0.0420	0.0419
**∆E** (**eV**)	0.4926	0.3373	0.3373	0.3124	0.3354	0.3126	0.2465	0.3124	0.3124	0.3126
**IP** (**eV**)	0.4649	0.2876	0.2875	0.2643	0.2928	0.2726	0.2912	0.2705	0.2704	0.2707
**EA** (**eV**)	−0.0277	−0.0497	−0.0498	−0.0481	−0.0426	−0.0400	0.0447	−0.0419	−0.0420	−0.0419
**χ** (**eV**)	0.2186	0.1189	0.1189	0.1081	0.1251	0.1163	0.1679	0.1143	0.1142	0.1144
**µ** (**eV**)	−0.2186	−0.1189	−0.1189	−0.1081	−0.1251	−0.1163	−0.1679	−0.1143	−0.1142	−0.1144
**η** (**eV**)	0.2463	0.1687	0.1687	0.1562	0.1677	0.1563	0.1233	0.1562	0.1562	0.1563
***S*** (**eV^−1^**)	4.0601	5.9294	5.9293	6.4016	5.9634	6.3980	8.1133	6.4016	6.4025	6.3977
**ω** (**eV**)	0.0970	0.0419	0.0419	0.0374	0.0467	0.0432	0.1144	0.0418	0.0418	0.0419
**µ** (**D**)	3.2995	6.4157	6.4206	3.3629	4.3809	2.5306	2.7504	2.6160	2.6316	2.7973

## Data Availability

The data presented in this study are available on request from the corresponding author.

## Supplementary Materials

The following supporting information can be downloaded at: https://www.mdpi.com/article/10.3390/molecules27248980/s1, Materials and Methods.

## Sample Availability

Samples of the compounds are available from the authors.
