# Supplementary material for: Mesomorphic, Computational Investigations and Dyeing Applications of Laterally Substituted Dyes
Source: Molecules. 2022 Dec 16;27(24):8980. doi: 10.3390/molecules27248980 (PMC9785732; doi:10.3390/molecules27248980)
Supplement: Supplementary file 1 [file molecules-27-08980-s001.zip › molecules-2028734-supplementary.pdf]

Supplementary data

Mesomorphic, Computational Investigations and Dyeing Applications of Laterally Substituted Dyes

Hoda A. Ahmed [1,2,*], Mohamed A. El-atawy [2,3], Fowzia S. Alamro [4], Nada s. Al-Kadhi [4], Omaima A. Alhaddad [5] and Alaa Z. Omar [3]

[1] Department of Chemistry, Faculty of Science, Cairo University, Cairo 12613, Egypt
[2] Chemistry Department, Faculty of Science, Taibah University, Yanbu, 46423, Saudi Arabia
[3] Chemistry Department, Faculty of Science, Alexandria University, P.O. 426 Ibrahemia, Alexandria, 21321, Egypt
[4] Department of Chemistry, College of Science, Princess Nourah bint Abdulrahman University, Riyadh 11671, Saudi Arabia
5 Chemistry Department, College of Sciences, Taibah University, Madina Monawara 30002, Saudi Arabia;
* Correspondence: ahoda@sci.cu.edu.eg (H.A.A.)

Characterizations

Melting points were determined by MEL-TEMP II melting point apparatus in open glass capillaries and were uncorrected. The IR spectra were recorded as potassium bromide (KBr) discs on a Perkin-Elemer FT-IR (Fourier-Transform Infrared Spectroscopy), college of Science, Taibah University. The NMR spectra were carried out at ambient temperature (~25 °C) on a (JEOL) 500 MHz spectrophotometer, NMR Unit, Faculty of Science, King Abdul-Aziz University. Chemical shift was recorded as δ values in parts per million (ppm), and the signals were reported as s (singlet), d (doublet), t (triplet) and m (multiplet). Elemental analyses were analyzed at the Micro analytical Unit, Faculty of Science, Cairo University. The electronic absorption spectra of the azo dyes 1-10 were measured by (pg instruments T80+) UV/visible spectrophotometer

TA Instruments Co. (Q20 Differential Scanning Calorimeter, DSC; USA) was used for

recording phase transitions. DSC calibration was carried using lead and indium melting temperature and enthalpy. Samples of 2–3 mg were used in aluminum pans for DSC investigation. The heating rate was 10°C/min in nitrogen gas as an inert atmosphere (30 ml/min). All transitions measured for the second heating scan.

Transition temperatures for the prepared compounds were checked and phases identified by Polarized optical microscope (POM, Wild, Germany) attached with Mettler FP82HT hot stage.

Dyeing process

The dyeing process of polyester fabrics was done in day bath of water and DYEWELL-002 as a levelling agent and dyeing accelerator which increases the diffusion property of disperse dyestuffs into the polyester fabrics and hence enables a good dyestuff pick-up. The pH of the dye bath was adjusted to 4-5 using aqueous acetic acid. Dyeing was done by raising the dye bath temperature to 130 °C under pressure in a dyeing machine at a rate of 3 °C/min, holding at this temperature for 60 min and then cooling to 50 °C. After dyeing, the polyester fabrics was washed and subjected to surface reduction clearing [(1 g NaOH + 1 g sodium hydrosulphite)/L then washed and air-dried.

Dye exhaustion

The consumption of the azo dyes by polyester fiber was measured by sampling the dye bath before and after the dyeing process. The dye bath concentration (g/l) was measured on (pg instruments T80+) UV/visible spectrophotometer at λmax of the appropriate dye.

Computational Method

All DFT calculations were performed using the Gaussian 09 software. A conformational search for obtaining the most stable conformer was done using the semi-empirical PM3 method. The most stable conformer was subjected to full geometrical optimizations using the DFT and Becke's three-parameter hybrid exchange functional in combination with the gradient-corrected correlation functional of Lee, Yang and Parr B3LYP/6-311G** method

without any constraints to calculations. All calculated structures were found to be true minima, i.e., no imaginary frequencies were observed after the ground state geometry optimization.

Synthesis of 4-((E)-((4-((E)-(2-chlorophenyl)diazenyl)naphthalen-1-yl)imino)methyl)phenol (1)

Step1: The chloroaniline (1 mmol) was dissolved in EtOH (3mL/mmol) und 2N HCl (1.5mL/mmol, 3.0equiv) was added. The mixture was cooled to 0°C and a pre-cooled solution of $NaNO_2$ (1.1equiv) in H_2O (1.5mL/mmol) was added dropwise over a period of 15min. After the end of the addition, the solution was stirred for further 15min at 0°C. Then the excess of $NaNO_2$ was removed by the addition of Sulfamic acid (0.2equiv) once again followed by stirring for 15 min at 0°C (solution A). The naphthylamine (1.0equiv) was dissolved in EtOH (20mL/mmol) and 10mL/mmol water was added (solution B). Solution A was then added to solution B at 0°C, the pH was determined of the resulting mixture (indicator paper) and it was stirred for 30 min at 0°C. Afterwards it was allowed to warm up to rt slowly followed by the addition of both DCM (20mL/mmol) and water (20mL/mmol). The aqueous phase was made alkaline (pH 10-12) by the addition of NaOH, and the product was extracted with DCM. The combined organic layers were dried over Na2SO4 and the solvent was removed under reduced pressure. The crude product was purified by crystallization from hot ethanol.

Step 2: Ethanolic solution of the intermediate azo compound (1 mmol) and 4-hydroxybenzaldehyde (1.1 mmol) in presence of few drops of acetic acid were heated under reflux for 2 h. drops of glacial acetic acid were added then cooled, filtered and finally the crude product was purified by crystallization.

Synthesis of 4-((4-alkoxyphenyl)diazenyl)naphthalen-1-ol (4)

A solution of 4-hexadecyloxyaniline (5.0 mmol) in hydrochloric acid was cooled in an ice-salt bath at 0-5°C. The amine solution was then added to a cold solution of sodium nitrite (5.2 mmol) at 0-5° C. The resulted mixture was stirred for 0.5h and the temperature was kept below 5°C. The formed diazonium salt solution was then added dropwise to a

aqueous solution of 1-naphthol/ NaOH (5.0 mmol/ 5.0 mmol). The resulted solution was vigorously stirred at 0-5 °C for 2 h, while the pH of the reaction mixture was maintained at 7-8 by simultaneous addition of potassium carbonate solution (0.5 M). The progress of the reaction was monitored by TLC and then a crude dye was filtered, washed with hot water for several times then recrystallized from water/ethanol mixture.

Synthesis of 4-((4-(hexadecyloxyl)phenyl)diazenyl)naphthalen-1-yl 4-substitutedbenzoate (8-10)

Molar equivalents of 4-((4-hexadecyloxyl)diazenyl)naphthalen-1-ol and the corresponding aromatic carboxylic acid namely, 4-octyloxybenoic acid, 4-decyloxyobenzoic acid or 4-hexadecyloxybenzoic acid (0.01 mole) were dissolved in 20 ml dry methylene chloride. N, N′-dicyclohexylcarbodiimide (DCCD) (0.02 mol) and few crystals of 4-dimethylaminopyridine (DMAP), as catalyst, were added. The solution left to stand for 70 hours at room temperature with continuous stirring. The byproduct, dicyclohexylurea (DCU), separated was then filtered off and the filtrate was then evaporated. The obtained solid residue was recrystallized twice from ethanol to give TLC pure products.

Synthesis of 2-fluoro-4-((4-(alkoxy)phenyl)diazenyl)phenol (2,3)

A solution of 4-alkoxyaniline (5.0 mmol) in hydrochloric acid was cooled in an ice-salt bath at 0-5°C. The amine solution was then added to a cold solution of sodium nitrite (5.2 mmol) at 0-5° C. The resulted mixture was stirred for 30 min. and the temperature was kept below 5°C. The formed diazonium salt solution was then added dropwise to an aqueous solution of 2-fluorophenol/ NaOH (5.0 mmol/ 5 mmol). The resulted solution was vigorously stirred at 0-5 °C for 2 h, while the pH of the reaction mixture was maintained at 7-8 by simultaneous addition of potassium carbonate solution (0.5 M). The progress of the reaction was monitored by TLC and then crude dyes was filtered, washed with hot water for several times and recrystallized from water ethanol mixture.

Synthesis of 2-fluoro-4-((4-alkoxy)phenyl)diazenyl)phenyl 4-(substituted) benzoate (5-7)

Molar equivalents of 2-fluoro-4-((4-(alkoxy)phenyl)diazenyl)phenol and 4-substitutedbenzoic acid (0.01 mol) were dissolved in 20 ml dry methylene chloride. *N*, *N′*-dicyclohexylcarbodiimide (DCC) (0.02 mol) and few crystals of 4-dimethylaminopyridine (DMAP), as catalyst, were added. The solution left to stand for 72 hours at room temperature with continuous stirring. The byproduct, dicyclohexylurea (DCU), separated was then filtered off and the filtrate was then evaporated. The obtained solid residue was recrystallized twice from ethanol to give TLC pure products.

***2-fluoro-4-((4-(octyloxy)phenyl)diazenyl)phenol* (2)**

Yield: 91.7 %; mp 123.0°C, FTIR (ύ, cm^{-1}): 3350 (OH), 2960, 2870 (CH2 stretching), 1594 (N=N), ^{1}H NMR (500 MHz,) δ 7.85 (d, *J* = 8.9 Hz, 2H, Ar-H), 7.64 (dd, *J* = 9.5, 4.5 Hz, 2H, Ar-H), 7.14 (t, *J* = 8.8 Hz, 1H, Ar-H), 6.98 (d, *J* = 8.8 Hz, 2H, Ar-H), 4.02 (t, *J* = 6.5 Hz, 2H, CH_2), 2.08 (s, exchangeable, 1H, OH), 1.85 – 1.77 (m, 2H, CH_2), 1.51 – 1.42 (m, 2H, CH_2), 1.39 – 1.23 (m, 10H, 5 CH_2), 0.89 (t, J = 6.9 Hz, 3H, CH3)., ^{13}C NMR (126 MHz,) δ 161.71(CH), 150.54 (C), 146.61(C), 146.05 (C), 124.72(CH), 122.38 (CH), 117.15 (CH), 114.83 (CH), 107.81 (CH), 107.66 (CH), 68.49 (CH_2), 31.92 (CH_2), 29.31 (CH_2), 26.20 (CH_2), 22.83 (CH_2), 14.27(CH_3). .Elemental Analysis Calc.(Found): C, 69.74 (69.72); H, 7.32 (7.25); N, 8.13 (8.10); F, 5.52 (5.48).

***2-fluoro-4-((4-(hexadecyloxy)phenyl)diazenyl)phenol* (3)**

Yield: 91.0 %; mp 112.0 °C, FTIR (ύ, cm^{-1}): 3355 (OH), 2950, 2865 (CH2 stretching), 1590 (N=N), ^{1}H NMR (500 MHz,) δ 7.85 (d, *J* = 8.9 Hz, 2H, Ar-H), 7.69 – 7.63 (m, 2H, Ar-H), 7.10 (t, *J* = 8.8 Hz, 1H, Ar-H), 6.97 (d, *J* = 8.9 Hz, 2H, Ar-H), 4.02 (t, *J* = 6.6 Hz, 2H, CH_2), 2.09 (s, exchangeable, 1H, OH), 1.84 – 1.75 (m, 2H, CH_2), 1.50 – 1.41 (m, 2H, CH_2), 1.38 – 1.19 (m, 24H, 12 CH_2), 0.86 (t, *J* = 6.9 Hz, 3H, CH_3). ^{13}C NMR (126 MHz,) δ 161.73, 146.63, 129.39, 124.74, 122.42, 117.12, 114.80, 114.59, 107.79, 107.64 , 68.54 (CH_2), 32.07 (CH_2), 29.82 (CH_2), 29.80 (CH_2), 29.66 (CH_2), 29.54 (CH_2), 29.43 (CH_2),

29.31 (CH_2), 26.15 (CH_2), 22.84 (CH_2), 14.30 (CH_3). Elemental Analysis Calc.(Found): C, 73.65 (73.60); H, 9.05 (8.88); N, 6.13 (6.12); F, 4.16 (4.15).

***2-fluoro-4-((4-(hexadecyloxy)phenyl)diazenyl)phenyl 4-fluorobenzoate* (6)**

Yield: 93.1 %; mp 103.0 $^{\circ}$C, FTIR (ύ, cm^{-1}): 2928, 2845 (CH_2 stretching), 1735 (C=O), 1586 (N=N), 1162 (C O $_{Ester}$), 1086 (C-O $_{Alkoxy}$). ^{1}H NMR (500 MHz,) δ 8.25 (dd, *J* = 8.8, 5.6 Hz, 1H, Ar-H), 7.91 (d, *J* = 9.0 Hz, 2H, Ar-H), 7.76 (ddd, *J* = 13.2, 9.9, 2.2 Hz, 2H, Ar-H), 7.40 (t, *J* = 8.2 Hz, 2H, Ar-H), 7.20 (t, *J* = 8.7 Hz, 1H, Ar-H), 7.00 (d, *J* = 8.8 Hz, 2H, Ar-H), 6.51 (d, *J* = 6.0 Hz, 2H, Ar-H), 4.04 (t, *J* = 6.7 Hz, 2H, OCH_2), 1.98 – 0.98 (m, 28H, 14 CH_2), 0.89 (t, *J* = 7.0 Hz, 3H, CH_3). ^{13}C NMR (126 MHz,) δ 167.53, 165.48, 163.16, 162.31, 156.91, 153.67, 151.65, 148.76, 146.55, 139.69, 139.53, 133.09, 125.20, 124.02, 121.01, 116.18, 116.00, 114.89, 109.09, 108.93, 106.69, 68.53 (CH_2), 49.23 (CH_2), 39.26 (CH_2), 35.02 (CH_2), 34.07 (CH_2), 31.93 (CH_2), 29.47 (CH_2), 29.29 (CH_2), 28.82 (CH_2), 26.00 (CH_2), 25.89 (CH_2), 25.55 (CH_2), 24.82 (CH_2), 22.81(CH_2), 14.25 (CH_3).Elemental Analysis Calc.(Found): C, 72.64 (72.63); H, 7.66 (7.62); N, 4.84 (4.79); F, 6.57 (6.57).

2-fluoro-4-((4-(hexyloxy)phenyl)diazenyl)phenyl 4-(decyloxy)benzoate (7)

Yield: 89.7 %; mp 69.0°C, FTIR (ύ, cm^{-1}): 2940, 2850 (CH_2 stretching), 1728 (C=O), 1590 (N=N), 1155 (C O $_{Ester}$), 1080 (C-O $_{Alkoxy}$). ^{1}H NMR (500 MHz,) δ 8.10 – 8.01 (m, 1H), 7.87 (d, *J* = 8.9 Hz, 2H), 7.58 (d, *J* = 9.1 Hz, 2H), 7.36 (dd, *J* = 12.0, 6.2 Hz, 1H), 7.12 – 7.09 (m, 1H), 6.99 – 6.97 (m, 2H), 6.85 (m, 2H), 5.53 (m, 2H), 3.99 (t, *J* = 6.5 Hz, 2H), 1.71 – 1.63 (m, 5H), 1.60 – 1.53 (m, 6H), 1.50 – 1.42 (m, 3H), 1.25 – 1.15 (m, 7H), 1.12 – 1.05 (m, 3H),103- 0.99 (m, 6H). Elemental Analysis Calc.(Found): C, 72.89 (72.87); H, 7.86 (7.85); N, 4.86 (4.84); F, 3.29 (3.28).

***4-((4-(Hexyloxy)phenyl)diazenyl)naphthalen-1-yl 4-(octyloxy)benzoate,* (8)**

Yield: 94.7%; mp 96.4°C, FTIR (ύ, cm^{-1}): 2925, 2848 (CH_2 stretching), 1730 (C=O), 1595 (N=N), 1160 (C O $_{Ester}$), 1080 (C-O $_{Alkoxy}$). ^1H NMR (500 MHz,) δ 8.99 (d, *J* = 8.5 Hz, 1H, Naph-H), 8.29 (d, *J* = 8.8 Hz, 2H, Naph-H), 8.05 (d, *J* = 8.8 Hz, 2H, Naph-H), 8.01 (d, *J* = 8.3 Hz, 1H, Ar-H), 7.89 (d, *J* = 8.2 Hz, 1H, Ar-H), 7.67 (d, *J* = 7.4 Hz, 1H, Ar-H), 7.61 – 7.54 (m, 1H, Ar-H), 7.46 (d, *J* = 8.5 Hz, 1H, Naph-H), 7.04 (m, 4H, Ar-H), 4.08 (m, 4H, OCH2), 3.72 (q, *J* = 6.9 Hz, 4H, CH_2), 1.88 – 1.80 (m, 4H, CH_2), 1.51 – 1.27 (m, 8H, CH_2), 1.24 (m, 4H, CH_2), 0.95 – 0.88 (m, 6H, CH_3). ^{13}C NMR (126 MHz,) δ 163.71(CO), 161.78(C), 148.81(C), 147.47(C), 132.49(CH), 132.42 (C), 132.33 (C), 127.54 (C), 127.04 (CH), 126.81 (CH), 125.06 (CH), 123.88 (CH), 121.43 (CH), 121.26 (C), 121.10 (C), 118.42 (CH), 114.75 (CH), 114.48 (CH), 111.61 (CH), 68.38 (OCH_2), 31.76 (CH_2), 31.58 (CH_2), 29.32 (CH_2), 29.21 (CH_2), 29.08 (CH_2), 29.05 (CH_2), 25.97 (CH_2), 22.66 (CH_2), 22.63 (CH_2), 22.60 (CH_2), 22.58 (CH_2), 18.39 (CH_2), 14.10 (CH_3), 14.03 (CH_3). Anal. Calc. for $C_{37}H_{44}N_2O_4$: C, 76.52; H, 7.64; N, 4.82. Found: C, 76.50; H, 7.61; N, 4.79.

4-((4-(Hexyloxy)phenyl)diazenyl)naphthalen-1-yl 4-(dodecyloxy)benzoate, **(9)**

Yield: 90.1%; mp 108.5 °C, FTIR (ύ, cm^{-1}): 2922, 2849 (CH_2 stretching), 1728 (C=O), 1598 (N=N), 1155 (C O $_{Ester}$), 1075 (C-O $_{Alkoxy}$). ^1H NMR (500 MHz,) δ 8.99 (d, *J* = 8.5 Hz, 1H, Naph-H), 8.29 (d, *J* = 8.8 Hz, 2H, Naph-H), 8.05 (d, *J* = 9.0 Hz, 2H, Naph-H), 8.01 (d, *J* = 8.5 Hz, 1H, Naph-H), 7.89 (d, *J* = 8.2 Hz, 1H, Ar-H), 7.69 – 7.64 (m, 1H, Ar-H), 7.58 (t, *J* = 7.5 Hz, 1H, Ar-H), 7.46 (d, *J* = 8.2 Hz, 1H, Ar-H), 7.05 (dd, *J* = 8.7, 6.2 Hz, 4H, Ar-H), 4.08 (td, *J* = 6.5, 3.3 Hz, 4H, OCH2), 1.88 – 1.80 (m, 4H, CH_2), 1.54 – 1.46 (m, 4H, CH_2), 1.41 – 1.35 (m, 6H, CH_2), 1.29 (d, *J* = 15.4 Hz, 14H, CH_2), 0.93 (t, *J* = 7.0 Hz, 3H,CH_3), 0.89 (t, *J* = 6.8 Hz, 3H, CH_3). ^{13}C NMR (126 MHz,) δ 163.78(CO), 161.80(C), 148.83(C), 147.61(C), 147.47(C), 132.49 (CH), 132.35 (C), 127.56 (C), 127.05 (CH), 126.82 (CH), 125.08 (CH), 123.90 (CH), 121.45 (CH), 121.29 (C), 121.12 (C), 118.43 (CH), 114.82 (CH), 114.48 (CH), 111.64 (CH), 68.39 (OCH_2), 31.90 (CH_2), 31.74 (CH_2), 31.36 (CH_2), 31.58 (CH_2), 29.62 (CH_2), 29.37 (CH_2), 29.14 (CH_2), 25.98 (CH_2), 25.87 (CH_2), 25.74 (CH_2), 22.68 (CH_2), 22.59 (CH_2), 14.13 (CH_3), 14.05 (CH_3). Anal. Calc. for $C_{41}H_{52}N_2O_4$: C, 77.32; H, 8.23; N, 4.40. Found: C, 77.31; H, 8.20; N, 4.39.

4-((4-(Hexyloxy)phenyl)diazenyl)naphthalen-1-yl 4-(hexadecyloxy)benzoate, **(10)**

Yield: 92.9%; mp 84.3 °C, FTIR (ύ, cm^{-1}): 2926, 2843 (CH_2 stretching), 1735 (C=O), 1598 (N=N), 1162 (C O $_{Ester}$), 1080 (C-O $_{Alkoxy}$). ^{1}H NMR (500 MHz,) δ 8.99 (d, *J* = 8.5 Hz, 1H, Naph-H), 8.29 (d, *J* = 8.8 Hz, 2H, Naph-H), 8.04 (d, *J* = 8.8 Hz, 2H, Naph-H), 8.01 (d, *J* = 8.4 Hz, 1H, Ar-H), 7.88 (d, *J* = 8.1 Hz, 1H, Ar-H), 7.69 – 7.62 (m, 1H, Ar-H), 7.58 (t, *J* = 7.5 Hz, 1H, Ar-H), 7.46 (d, *J* = 8.4 Hz, 1H, Naph-H), 7.04 (m, 4H, Ar-H), 4.08 (m, 4H, OCH_2), 1.88 – 1.81 (m, 4H, CH_2), 1.54 – 1.46 (m, 4H, CH_2), 1.41 – 1.34 (m, 4H, CH_2), 1.30 – 1.23 (m, 24H, CH_2), 0.93 (m, 3H, CH_3), 0.88 (t, *J* = 6.9 Hz, 3H, CH_3). ^{13}C NMR (126 MHz,) δ 163.82(CO), 161.94(C), 148.97(C), 147.63 (C), 132.64 (CH), 132.56 (C), 132.49 (C), 128.92 (CH), 127.70 (C), 126.97 (CH), 125.23 (CH), 124.05 (CH), 122.21 (CH), 121.59 (CH), 121.26 (C), 118.58 (CH), 114.90 (CH), 114.64 (CH), 111.77 (CH), 68.54 (OCH_2), 32.06 (CH_2), 31.93 (CH_2), 31.83 (CH_2), 31.74 (CH_2), 29.82 (CH_2), 29.79 (CH_2), 29.73 (CH_2), 29.64 (CH_2), 29.52 (CH_2), 29.38 (CH_2), 29.29 (CH_2), 29.21 (CH_2), 26.13 (CH_2), 25.90 (CH_2), 22.83 (CH_2), 22.74 (CH_2), 14.28 (CH_3), 14.19 (CH_3). Anal. Calc. for $C_{45}H_{60}N_2O_4$: C, 78.00; H, 8.73; N, 4.04. Found: C, 77.98; H, 8.71; N, 4.01.
